# Effect of physical therapy on breast cancer related lymphedema: protocol for a multicenter, randomized, single-blind, equivalence trial

**DOI:** 10.1186/1471-2407-14-239

**Published:** 2014-04-03

**Authors:** Mette Tambour, Berit Tange, Robin Christensen, Bibi Gram

**Affiliations:** 1 Department of Physical Therapy, Hospital of Southwest Jutland, Esbjerg, Denmark; 2 Musculoskeletal Statistics Unit, The Parker Institute, Department of Rheumatology, Copenhagen University Hospital, Bispebjerg and Frederiksberg, Copenhagen, Denmark; 3 Institute of Sport Science and Clinical Biomechanics, University of Southern Denmark, Odense, Denmark; 4 Center for Clinical Research, Hospital of Southwest Jutland, Esbjerg, Denmark; 5 Institute of Regional Health Research, University of Southern Denmark, Odense, Denmark

**Keywords:** Manual lymphatic drainage, Complete Decongestive Therapy, Breast cancer

## Abstract

**Background:**

Physical therapy treatment of patients with lymphedema includes treatment based on the principles of ‘Complete Decongestive Therapy’ (CDT). CDT consists of the following components; skin care, manual lymphatic drainage, bandaging and exercises. The scientific evidence regarding what type of treatment is most effective is sparse. The objective of this study is to investigate whether CDT is equally effective if it includes manual lymphatic drainage or not in the treatment of arm lymphedema among patients with breast cancer.

**Methods/Design:**

A randomized, single-blind, equivalence trial. A total of 160 breast cancer patients with arm lymphedema will be recruited from 3 hospitals and randomized into one of two treatment groups A: Complete Decongestive Therapy including manual drainage or B: Complete Decongestive Therapy without manual lymphatic drainage. The intervention period will be approximately 4 weeks followed by a 6 month follow-up period (7 months from baseline). Primary outcome variable: the percentage volume reduction of lymphedema (%) from baseline to 7 months. Secondary outcome variables: Differences from baseline to week 4 and from week 4 to month 7 in circumference of the arm (cm), body weight (kg), patient sensation of heaviness (scale range: 0–10), patient sensation of tension (scale range: 0–10), and quality of life (EQ-5D-5 L-questionnaire).

All measurements are standardized and will be performed before randomization, after 4 weeks and after 7 months.

**Discussion:**

This randomized controlled study seeks to provide data on an effective treatment for patients with breast cancer related arm lymphedema and which at the same time causes minimal patient inconvenience.

**Trial registration:**

ClinicalTrials.gov: Identifier NCT02015897

## Background

Breast cancer is the most common type of cancer in women and in 2011, 4607 women in Denmark were diagnosed with breast cancer [1]. One of the well-known complications of breast cancer treatment is secondary lymphedema; an accumulation of protein-rich interstitial fluid due to the insufficient capacity of the lymphatic system. Axillary lymph-node dissection and subsequent radiation therapy are the most common causes of arm lymphedema [2,3]. The incidence of lymphedema as a sequelae to breast cancer treatment ranges from 6-50%, depending on the surgical procedure in the axilla and the type of the radiation therapy [2,4]. The reported prevalence relies among other things on the definition of lymphedema and which techniques are used to measure it [5]. Lymphedema occurs most often within the first year following the treatment [6,7] and 77% of the patients develop lymphedema within the first three years post-surgery [8].

Lymphedema is a chronic condition and when untreated, the risk of worsening of the lymphedema over time in terms of volume and stage of tissue fibrosis increases [9-11]. In addition to the cosmetic deformities, lymphedema causes impaired physical mobility, mental discomfort, social isolation [12], reduced quality of life [12,13] and higher incidence of erysipelas [12,14].

Physical therapy treatment of patients with lymphedema is based on the principles of ‘Complete Decongestive Therapy’ (CDT). CDT consists of the following components; skin care, manual lymphatic drainage, bandaging and exercises. Traditionally, the bandages used in CDT have been short-stretch bandages. In Denmark, the most commonly used manual lymphatic drainage method is described by Földi [15-17]. The treatment is based on manual lymphatic drainage 4–5 times weekly and each treatment takes 30–45 minutes. However, standardization between different treatment locations and among treating physiotherapists does not exist.

The scientific evidence regarding what type of treatment or combination of treatments is most effective is sparse. Studies [2,3,7,14] have focused on time-consuming manual lymphatic drainage, but the scientific evidence is not consistent [18-20]. The lack of robust evidence may be a contributory cause to the variation of available treatment options among different treatment locations and among treating physiotherapists.

This includes both the combination of treatment methods used and the time spent on each treatment. Furthermore, the type of bandages used varies. Traditionally, the patients receive the manual lymphatic drainage in combination with the replacement of the bandage i.e. 4–5 times weekly, but a new type of bandage may only require replacement two or three times weekly [21]. Reduced replacement of the bandage leads to fewer manual lymphatic drainage treatments and it is unknown whether use of the relatively new but fairly common bandage, Coban™2 Lite, alone can constitute the treatment.

In a recent systematic review [18], the authors concluded that there was not sufficient scientific evidence supporting the use of manual lymphatic drainage in preventing or treating lymphedema. Furthermore, the authors found that the methodological quality of the studies reviewed was poor and with clinical and statistical inconsistencies between the various studies. Therefore, there is a need for well-designed, high quality studies with follow-up.

The hypothesis of this study is that skin care and guidance of physical activity combined with Coban™2 Lite bandage alone is equally effective as Complete Decongestive Therapy including skincare and guidance of physical activity, manual lymphatic drainage and Coban™2 Lite bandage.

### Objectives

Complete Decongestive Therapy is equally effective whether it includes manual lymphatic drainage or not in the treatment of arm lymphedema among patients with breast cancer.

## Methods/Design

### Trial design

**Figure 1:**
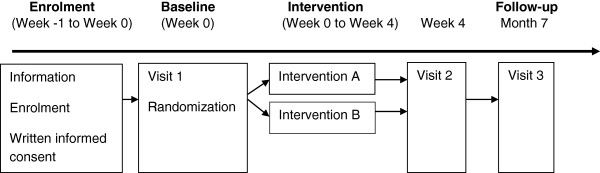
Flow-chart: equivalence trial – lymphedema study.

The study is a randomized, single blinded, equivalence trial aiming to determine whether an applied composite treatment is equally good if one of its components, manual lymphatic drainage, is removed. The design is illustrated in Figure 1.

The intervention period is approximately 4 weeks followed by 6 months follow-up period. Overall length of the study is 7 months. Primary endpoint will be 7 months from baseline.

The patients complete baseline measurements before the randomization.

### Participants

Patients with breast cancer related lymphedema will be recruited from the Hospital of Southwest Jutland, Lillebaelt Hospital, and the Hospital of Southern Jutland. The following eligibility criteria will be used to determine if a patient can be included:

Inclusion criteria

• Breast cancer diagnosis regardless of the date of operation and identified lymphedema

• Ultrasound scanning of the axilla in order to exclude local relapse

• Lymphedema > 2 cm and stage II-III [22]

• Completed radiotherapy and/or chemotherapy at least 2 months prior to inclusion

Exclusion criteria

• Relapse of breast cancer

• Untreated infection

• Untreated heart failure

• Untreated renal failure

• Untreated deep venous thrombosis in the arm

• Inability to participate in physical therapy treatments and/or inability to understand the instructions

### Intervention

#### Treatment groups

The treatment of the patients will be conducted at the departments of physical therapy, Hospital of Southwest Jutland (Esbjerg), Lillebaelt Hospital (Vejle), and the Hospital of Southern Jutland (Aabenraa). The treatment will be performed by lymphedema physiotherapists.

The number of patients included in the study is expected to be equally distributed between the 3 centers. The patients will be randomized into two treatment groups at each treatment site. The intervention period for both groups will be minimum 2 weeks. In approx. three weeks, the lymphedema is expected to be treated to a stable condition estimated by circumference measurements. The circumference of the arm will be measured at seven different locations on the arm at each treatment (twice a week). When two consecutive measurements are stationary (< 0.5 cm difference in three out of five measurements), the active treatment period will end and a permanent and individually tailored compression sleeve/garment will be ordered. The treatment will continue until the sleeve is made and can be given to the patient.

##### Treatment group A

The treatment group A will receive CDT-treatment as offered today at the Hospital of Southwest Jutland, Esbjerg. The treatment includes:

• Skin care

• Manual lymphatic drainage

• Bandaging using Coban™2 Lite

• Guidance on physical activity

The patients will receive the manual lymphatic drainage for 30 minutes twice a week. Total treatment time for each CDT-treatment session will be one hour.

##### Treatment group B

Treatment for group B includes:

• Skin care

• Bandaging using Coban™2Lite

• Guidance on physical activity

Treatment time for each session will be 30 minutes and will be given twice a week.

All measurements and questionnaires will be performed before randomization, after approximately 4 weeks and after 7 months (Table 1). The measurements will be performed by non-treating personnel.

**Table 1:** Outcomes and variables assessed during the trial period

**Variable**	**Baseline visit 1**	**4 weeks visit 2**	**7 months visit 3**
Age	X		
Body height,cm	X		
Arm lymphedema volume, ml	X	X	X
Circumference of the arm, cm	X	X	X
Body weight, kg	X	X	X
Sensation of heaviness, score	X	X	X
Sensation of tension, score	X	X	X
Quality of life (EQ-5D-5L), score	X	X	X

#### Specification of each treatment component

##### Skin care

In order to maintain the integrity of the skin, at each treatment the arm is bathed and treated with softening and unscented lotion.

##### Manual lymphatic drainage

Manual lymphatic drainage aims to remove excess interstitial fluid and increase lymphatic transport. The treatment method used is according to descriptions by Földi [15-17]. The manual lymphatic drainage is a gentle massage and the movements are slow and rhythmical. The method consists of 4 basic techniques with a pressure phase and a relaxation phase.

##### Bandaging

Coban™2 Lite is a two-component compression system with a comfort layer close to the skin and a self-adherent external compression layer. The comfort layer is applied with minimal overlap and without tension whereas the compression layer is applied with 50% overlap and at full stretch, corresponding to a pressure of approximately 30 mmHg. The bandage is retained until the next treatment.

##### Guidance on physical activity

In order to maintain/increase the mobility, the patients are encouraged to maintain normal function and information about the muscle pump is provided.

### Guidance of sleeve treatment in the observation period

The sleeve is considered to be a permanent assistive device and when given to the patients, they will be instructed in applying the sleeve. Additionally, it will be recommended that they keep the sleeve on every day from morning till night.

### Study endpoints and assessments

*Primary outcome measure*: The percentage volume reduction of arm lymphedema (%) from baseline to 7 months.

*Secondary outcome measure*: Differences in circumference of the arm (cm), bodyweight (kg), patient sensation of heaviness (scale range: 0–10), patient sensation of tension (scale range: 0–10), and quality of life (EQ-5D-5 L-questionnaire).

Percentage volume reduction of arm lymphedema (%) from baseline to week 4.

Percentage volume reduction of arm lymphedema (%) from week 4 to week 7.

Length of treatment period until stable circumference of the arm (days).

All measurements are standardized and will be performed before randomization, after approximately 4 weeks and after 7 months (Table 1).

### Measurements

*Arm volume* (*ml*): Inverse Water Displacement Volumetry method [23] using Bravometer (Novuqare BV, PJ Horst, NL).

*Circumference* (cm) of the arm using measuring tape: Starting from the wrist, 5 circumference measures will be noted: wrist (1 measure) forearm (2 measures) and upper arm (2 measures) [24].

*Patient sensation of heaviness*: Scale ranging from 0–10 (0: no heaviness, 10: worst imaginable heaviness).

*Patient sensation of tension*: Scale ranging from 0–10 (0: no tension, 10: worst imaginable tension).

*Quality of life*: EQ-5D-5 L questionnaire (http://www.euroqol.org) [25].

### Sample size

The margin of equivalence, Δ, was defined as less than 12% points and the range -12% to +12% points thus is predefined as an acceptable range of imprecision (i.e., 95% confidence interval). This margin is based on clinically and statistically important differences as well as ethical criteria, cost and feasibility. In a two one-sided tests (TOST) analysis for additive equivalence of two-sample normal means with bounds -12 and 12 for the mean difference and a significance level of 0.05, assuming a null difference and a common standard deviation of 25%, a sample size of 76 patients with arm lymphedema per group is required to obtain a power of at least 0.8. It was decided to enroll 160 patients in the intention-to-treat (ITT) population. Secondarily to explore a potential difference between the groups, including 160 patients with a two-sided significance level of 0.05, assuming a common SD of 25%, a sample size of 80 per group has a power of 85.5% to detect a mean difference of 12% points.

### Randomization and allocation concealment

After the baseline assessment, the participants will be randomly assigned to either treatment A or treatment B. The randomization sequence will be created using SAS (SAS 9.2) statistical software stratifying patients by center with a 1:1 allocation using random block sizes of 2, 4 and 6. The allocation sequence will be concealed from the researcher enrolling and assessing participants in sequentially numbered, opaque, sealed and stapled envelopes. Aluminum foil inside the envelope will be used to render the envelope impermeable to intense light.

The patients will be randomized (1:1) in one of the 2 groups:

1. Treatment-group A: standard treatment offered at the hospital containing skincare, manual lymphatic drainage, bandaging and guidance on physical activity.

2. Treatment-group B: standard treatment except for the manual lymphatic drainage. The treatment will include skin-care, bandaging and guidance on physical activity.

### Statistical analyses

All data analyses will be carried out according to a pre-established analysis plan and all analyses will be done using SAS software (v. 9.2; SAS Institute Inc., Cary, NC, USA). All descriptive statistics and tests will be reported in accordance with the CONSORT statement for non-inferiority/equivalence trials [26], which is the recommendation of the “Enhancing the QUAlity and Transparency Of health Research” (EQUATOR) network [27]. In order to evaluate the empirical distributions of the continuous outcomes, visual inspection of the studentized residuals will be applied to evaluate whether the assumption of normality is reasonable.

Baseline demographic and clinical characteristics will be reported descriptively for all patients in the full analysis set.

The primary analyses will be based on the ‘full analysis set’ , referred to as the intention-to-treat (ITT) population. Missing data following baseline measurements will be replaced using a non-responder imputation (i.e., baseline observation carried forward) [28]. For assessments of change from baseline, analyses of covariance (ANCOVA) will be applied, with treatment assignment serving as the main factor and the baseline value as covariate. Point estimates and 95% confidence intervals (95% CIs) will be provided for the difference in adjusted mean change from baseline between the two treatment groups.

For the discrete (dichotomous) data, the estimate of the proportion of responders (with 95% CIs) within each treatment group, as well as the 95% CIs for the difference in response rates between treatment groups, will be presented.

Equivalence approach to analysis set: In order to demonstrate equivalence the entire 2-sided 95% CI lies within -12% points and +12% points. Thus, the per-protocol population, which includes the subset of patients from the ITT population who had no relevant protocol deviations, will be used to confirm the primary efficacy (equivalence) end point.

### Ethics

Prior to inclusion in the study, all participants will receive written and oral information about the study and informed consent will be obtained from all participants before enrollment in the study. The participants are allowed to withdraw from the project without any further explanations or consequences. Patients who do not wish to participate in the study will receive the standard hospital treatment of lymphedema.

The hospital’s doctors refer patients with lymphedema to physical therapy. At the first meeting with the physiotherapist, patients who are assessed to be in need of Complete Decongestive Therapy will be informed about the study. Written materials will be handed out at the first meeting and it will be emphasized to the patient that at the next appointment, if needed, there will be the opportunity to be accompanied by an observer. A written informed consent will be obtained at the latest on the third appointment or within a week from the first contact.

The study is registered with *The Danish Data Protection Agency*, and in *ClinicalTrials.gov* (NCT02015897) http://www.clinicaltrials.gov, and approved by *The Regional Scientific Ethical Committee for Southern Denmark* (S-20130091). Participation in the study is not considered to be hazardous to the patient and no side effects associated with treatment are expected.

## Discussion

In clinical practice it is important to investigate the methods used and elucidate those which are most effective. Several studies [18] have been published focusing on the effect of Complete Decongestive Therapy (CDT), an often used method in the treatment among breast cancer patients with arm lymphedema. Manual lymphatic drainage is a time-consuming component in CDT. However, there is not sufficient scientific evidence supporting the use of manual lymphatic drainage in preventing or treating arm lymphedema. Moreover, there is focus on the issue of resources; how we can identify the most effective arm lymphedema treatment and achieve the desired clinical benefit with minimal patient inconvenience.

This study aims to investigate if prioritizing the well-known components in the CDT-treatment differently can provide equally good effect. All treatment methods used in the study are a part of everyday clinical practice.

The participants will be recruited from and treated at three hospitals in Denmark located in the region of Southern Denmark. This setting will enable a bigger sample size and with 7 months follow-up it will be possible to include long term monitoring. Additionally, the study may, in the long term, lead to a standardized method in the treatment of arm lymphedema.

This study will contribute to setting more focus on scientific decision making in clinical practice. The knowledge from this study can be implemented into the treatment of the patients since the study is based on an approach used in the clinical setting.

The results will be disseminated to all groups with a vested interest including (1) the scientific and professional community; (2) the research participants, and (3) the general public.

## Competing interests

The authors declare that they have no competing interests.

## Authors’ contributions

MT, BT, RC, and BG: Drafted the manuscript. MT, RC, and BG: Designed the study. RC and BG: power and sample size calculation and describing the statistical analysis as well as the allocation and randomization procedure. All authors read and approved the final manuscript.

## Pre-publication history

The pre-publication history for this paper can be accessed here:

http://www.biomedcentral.com/1471-2407/14/239/prepub
